# Magmatic overpressures, volatile exsolution and potential explosivity of fissure eruptions inferred via dike aspect ratios

**DOI:** 10.1038/s41598-020-66226-z

**Published:** 2020-06-10

**Authors:** Nobuo Geshi, John Browning, Shigekazu Kusumoto

**Affiliations:** 1 Research Institute of Earthquake and Volcano Geology, Geological Survey of Japan, National Institute of Advanced Industrial Science and Technology, Tsukuba, Japan; 2 Department of Mining Engineering and Department of Structural and Geotechnical Engineering, Pontificia Universidad Católica de Chile, Santiago, Chile; 3 Andean Geothermal Centre of Excellence (CEGA), Santiago, Chile; 4 Graduate School of Science and Engineering for Research, The University of Toyama, Toyama, Japan

**Keywords:** Natural hazards, Solid Earth sciences

## Abstract

Buoyant magmas abundant in exsolved volatiles (bubbles) drive the rapid upward-propagation of feeder dikes from magma chambers. The consequence of a feeder dike reaching the surface can result in an explosive volcanic eruption depending, partly, on the retention of volatiles. Therefore, timely detection of the vesicularity and overpressure of the magma during feeder dike ascent is critical for the prediction of the explosivity of any future eruption. In this study, we evaluated the explosivity of eruptions based on field investigations of the erupted products and the overpressure of magma in the conduit based on the dimensions of exposed feeder dikes. We found a positive correlation between the explosivity of eruptions and the magma overpressure generated in the conduit during recent fissure eruptions of Miyakejima volcano. Because the buoyancy of low-density magma produces positive overpressure at the dike’s top, feeder dikes with highly-vesiculated magmas possess high amounts of overpressure. An enlargement of the opening width of a feeder dike by magmatic overpressure results in a higher flux of vesiculated magma, which causes vigorous explosive activity. Our results suggest the possibility of forecasting the explosivity of an impending eruption if the width (or opening) of an ascending feeder dike is monitored in real-time through measurements of ground deformation and seismicity induced by the dike.

## Introduction

Magmatic overpressure is the main driving force of magma discharge through a conduit. The explosivity of an eruption is, in the simplest case, controlled by the discharge rate of magma containing pressurized bubbles through the conduit. As magmatic overpressure causes elastic deformation in the surrounding host rock during the growth of an intruding dike^1,2^, we can evaluate the magnitude of the overpressure using the intensity of the ground deformation induced by a dike^3–5^. It may also be possible to evaluate the explosivity of an impending eruption based on real-time monitoring of the ground deformation and seismic activity induced by the intruding dike. In order to plan the response to a volcanic crisis and, hence, manage the hazard, an evaluation of the potential explosivity of an impending fissure eruption is crucial^6,7^. However, the relationship between the overpressure and vesicularity in a feeder dike and the explosivity of an eruption fed by a dike has not, so far, been examined with a field-based dataset. This is because only a select few volcanoes can provide such a specific dataset that demonstrates both the explosivity of eruptions and the overpressure in the conduit.

In this study, we evaluated the effect that magma overpressure within a feeder dike had on the explosivity of an eruption. Miyakejima volcano is a rare case that provides a full dataset of the explosivity of eruptions, the dimensions of feeder dikes beneath the vents, and information concerning the approximate depth to the feeding magma chambers. A collapse caldera formed during the 2000 AD eruption of Miyakejima, subsequently truncated several historical eruption fissures, and exposed their feeder dike systems^8,9^. In combining datasets from the eruptive products and the geometric data of the feeder conduit, we explored the relationship between the explosivity of fissure eruptions and the magmatic overpressure and vesicularity in their feeder dikes.

The Miyakejima volcano is an active stratovolcano with basaltic–andesitic compositions. The ground-deformation pattern and petrological evidence from the erupted magmas suggest that the depth to an andesitic magma chamber is ~5 km and to a basaltic magma chamber is ~10 km beneath the volcanic edifice^10^. Miyakejima has experienced many fissure eruptions from predominantly radial dikes. Comparing to these depths of magma chambers and the horizontal distribution of fissure eruptions on the island, which are within ~4 km from the center of the volcanic edifice, the feeder dikes for these fissure eruptions must have propagated sub-vertically from the magma chambers, though additional lateral propagation of dikes to areas outside of the island was also interpreted during the 2000 AD eruption^11^

## Field occurrence of feeder dikes

**Figure 1:**
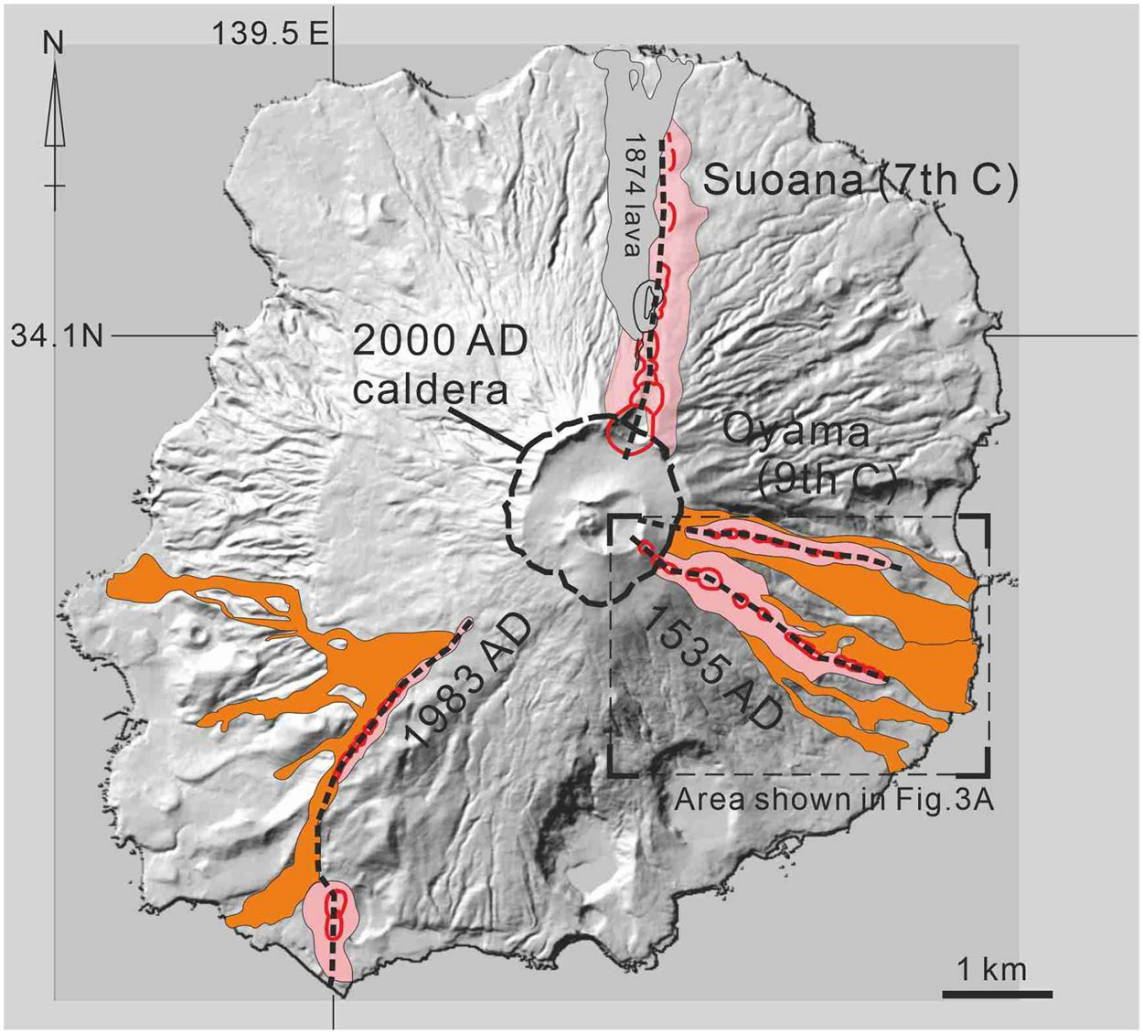
Distribution of four fissure eruptions and their eruptive products. Broad black lines show the location of eruption fissures. The pink-colored areas and orange-colored areas in Figure A show the distribution of the scoria cone deposits and lava flows ejected from the eruption fissures, respectively. Location of the rim of the 2000 AD caldera is shown by  a broken line. The relief map was created from the 10-m-mesh DEM of the Hokkaido Chizu Co.Ltd. based on the topographic map before the 2000 eruption.

**Figure 2:**
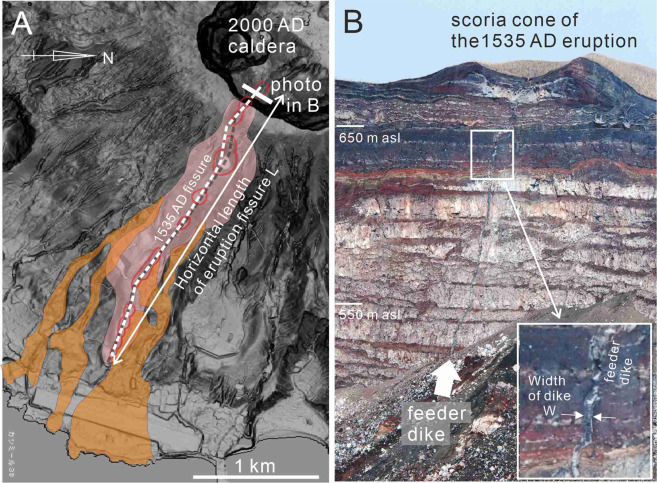
Distribution of the 1535 AD eruption fissure on the ground surface (Fig. A) and the outcropping feeder dikes (Fig. B) of the 1535 AD eruption. The white broken line in Figure A shows the trace of the eruption fissure of the 1535 AD eruption. Red lines show the craters. Pink-colored area and orange-colored area in Figure A show the distribution of the scoria cone deposit and lava flow ejected from the eruption fissure, respectively. Figure B show the feeder dike and the cross section of scoria cone of the 1535 AD eruption exposing on the wall of the 2000 AD caldera. The location of this outcrop is shown in Figure A. The feeder dike on the caldera wall in Figure B is indicated by white arrow.

We select four fissures (Fig. 1) that offered the most complete exposure of both their feeder dikes and the eruption fissures (Fig. 2 and Supplementary File). These include the feeder dikes of the Suoana eruption in the 7^th^ century^12^, the Oyama eruption in the 9^th^ century^13,14^, and the eruptions in 1535^13,14^ and 1983 AD^15,16^. Parameters of dike geometry (horizontal length L and width w of a feeder dike) and petrological characteristics (e.g. chemical composition and vesicularity of the magma) are obtained from these eruption fissures (Table 1).

**Table 1:** Parameters of analysis.

	Eruption type	whole-rock SiO_2_ (wt%)	Minimum vesicularity (vol.%)	Horizontal length of dike: L (m)	Width of the dike: w (m)	Depth to the magma chamber: h (m)	Density of the host rock (ρ_m_ (kg/m^3^)	P wave velocity: V_p_ (m/s)	S wave velocity: V_s_ (m/s) (V_s_ = 0.57V_p_)	Young’s modulus: E (GPa)	Poisson’s ratio ν	Dike aspect ratio: w/L (× 10^4^)	Calculated overpressure in the dike: P_o_ (× 10^6^ Pa)	Calculated vesicularity in the dike: p (%) when Pe = 2 × 10^6^Pa	Calculated vesicularity in the dike: p (%) when Pe = 0 Pa
Oyama eruption	Lava effusion^14^	53.3 ± 0.8	<1	2400	0.8^14^	10000	2700	3300 ± 330^18^	1881 ± 188	24	0.25	3.3	4.2 ± 0.8	0.8 ± 0.3	1.6 ± 0.3
1983AD eruption	Lava effusion with weak fountaining^14^	54.3 ± 1.0	~1–2	4000	0.8^16^	5000	2700	3300 ± 330^18^	1881 ± 188	24	0.25	2.0	2.5 ± 0.9	0.4 ± 0.4	1.9 ± 0.4
1535 AD eruption	Lava effusion with weak fountaining^14^	52.1 ± 0.3	<1	3000	1.1^14^	10000	2700	3300 ± 330^18^	1881 ± 188	24	0.25	3.7	4.7 ± 0.9	1.0 ± 0.4	1.8 ± 0.4
Suoana eruption (Phase I)	Vigorous fire fountainig^14^	60.6 ± 0.7	~10	2800	3.5^14^	5000	2650	3300 ± 330^18^	1881 ± 188	23	0.25	12.5	13 ± 3	10.5 ± 2.4	12 ± 2.4

We regard the horizontal length of the eruption fissure (Fig. 2A) as the horizontal length of the feeder dike The measured horizontal length of the eruption fissures is 2.8 km for the Suoana eruption, 2.3 km for the Oyama eruption, 2.8 km for the 1535 AD eruption, and 4.0 km for the 1983 eruption. We regard the width of feeder dike as the average width of the exposed feeder dike on the outcrop (Fig. 2B). The feeder dikes of the eruptions of the Suoana, Oyama and the 1535 AD eruption are exposed on the wall of the 2000 AD caldera^9^. The feeder dike of the 1983 AD eruption is exposed on an explosion crater which was formed during the 1983 eruption^16^. The average width of the feeder dikes (w) in the outcrops are 3.5 m, 0.8 m, and 1.1 m, respectively, for the Suoana, Oyama, and 1535 AD eruptions^14^ and 0.8 m for the 1983 AD eruption^16^.

The explosivity of these eruptions can be estimated from the erupted materials distributed around the eruption fissures. Vigorous fire fountains during the Suoana eruption produced thick piles of agglutinate bed which can be traced up to 300 m from the eruption fissure^12^. Conversely, developments of agglutinates around the Oyama, the 1535 and the 1983 eruptions fissures are limited in areas within ~100 m from the fissures. Most of the magmas erupted during these eruptions are produced as lava flow, though fairly high fire fountains were observed at the opening phase of the 1983 eruption^15^. During the 1983 eruption, pyroclastic materials occupy only 7.5% of the erupted magma from the inland vents where phreatomagmatic eruptions did not occur, and the remaining 92.5% erupted as lava flow^15^. These observations indicate a relatively gentler and effusive feeding of lava flows with weak explosive activity^14,15^. This qualitatively indicates that the Suoana eruption had a somewhat higher volatile content than the other three eruptions.

The chilled or glassy margins of a dike can freeze the vesicularity of a magma, as the magma does not have sufficient time to outgas before being quenched. The feeder dikes of the 1983 AD eruption have dense chilled margins with 1–2 vol.% of vesicularity; however, the central portion of the feeder dike possesses a much higher vesicularity of up to 40%^16^, owing to the secondary vesiculation of the dike interior. The semi-vertical wall of the 2000 AD caldera prevents access to the feeder dikes of the Suoana, Oyama, and 1535 AD eruptions, so their vesicularities are unknown. However, dense and glassy juvenile bombs found within the proximal deposits of their eruption fissures suggest the vesicularity of magmas in the conduit. This is because the vesicularities were quenched through contact with external water before any secondary vesiculation after ejection. The dense and glassy bombs of the Oyama and 1535 AD eruption fissures have vesicularities of less than 1 vol. %. The minimum vesicularity of the dense and glassy bombs of the Suoana eruption is around 10 vol.%.

## Magma overpressure in dike

**Figure 3:**
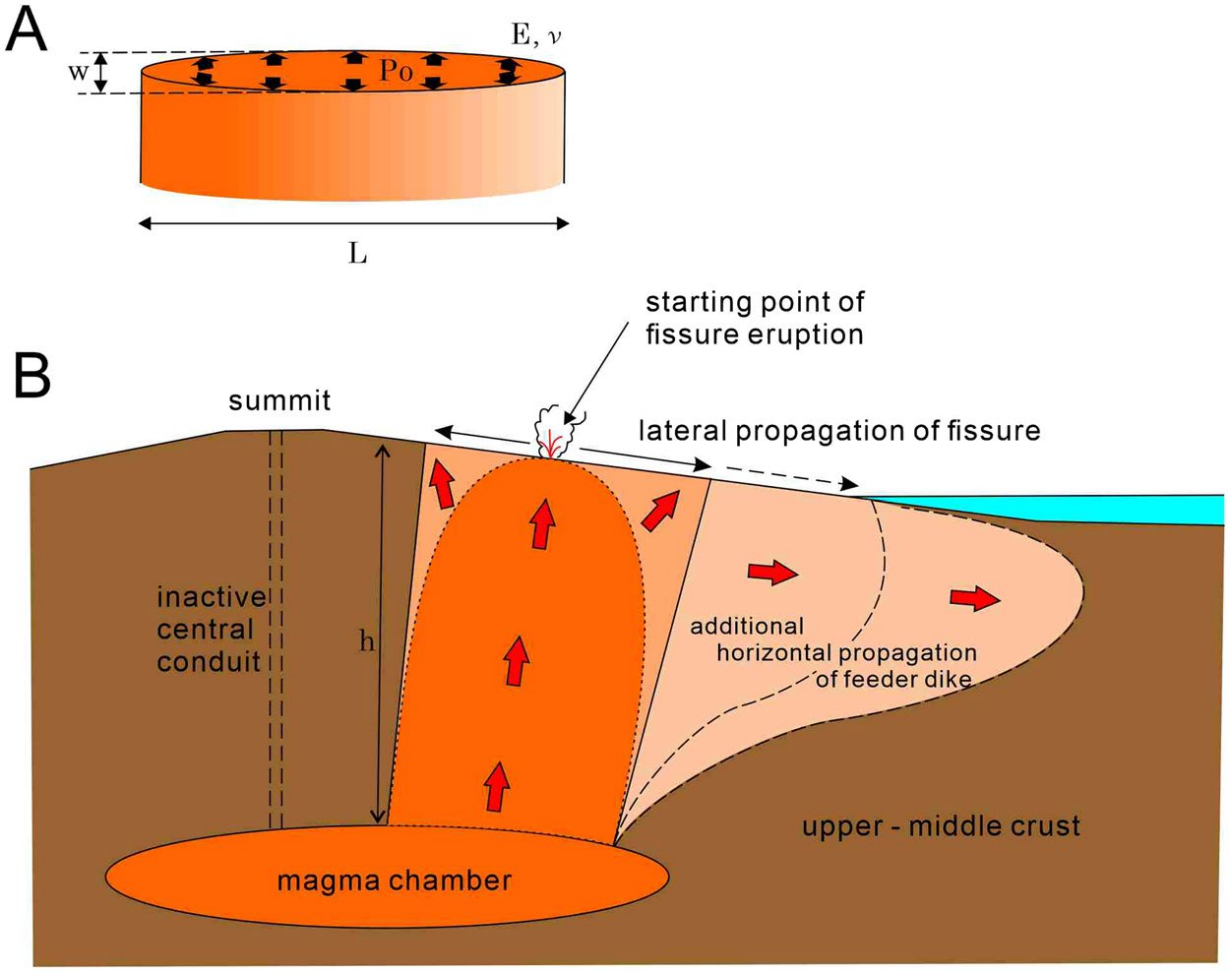
(**A**) Illustration of the geometry of typical feeder dike, where w is the opening or thickness of the dike and L is the along strike length of the dike. Po is the overpressure of magma in the dike. E and ν is the Young’s modulus and Poisson’s ratio of the host rock, respectively. (**B**) Schematic cross section along an eruption fissure. Height of the feeder dike (h) is the depth to the magma chamber. Fissure eruption starts when a feeder dike intersects the ground surface. The fissure eruption will propagate in both directions as the growth of feeder dike progresses. When the feeder dike changes direction horizontally, the eruption fissure can propagate laterally.

Magmatic overpressure in a dike is defined as a difference between the magmatic pressure inside a dike and the lithostatic pressure in the surrounding host rock. The relationship between the overpressure P_o_ of magma filling a dike in an elastic rock and the dike aspect ratio w/L of a two-dimensional elliptical crack is given as follows:

1 $${{\boldsymbol{P}}}_{{\boldsymbol{o}}}=\frac{{\boldsymbol{wE}}}{2{\boldsymbol{L}}(1-{{\boldsymbol{\nu }}}^{2})}$$

where L is the length of the dike, and w is the maximum opening width of the dike at the dike center (Fig. 3). E is Young’s modulus and ν is Poisson’s ratio of the surrounding rock^1,17^. We replaced Young’s modulus E with shear modulus G as per Eq. (2):

2 $${\boldsymbol{E}}=2{\boldsymbol{G}}(1+{\boldsymbol{\nu }})=2(1+{\boldsymbol{\nu }}){{\boldsymbol{\rho }}}_{{\boldsymbol{r}}}{{\boldsymbol{V}}}_{{\boldsymbol{s}}}^{2}$$

where ρ_r_ is the density of the rock and V_s_ is the velocity of S-wave in the rock. Young’s modulus in Eq. (2) is dynamic. Combining Eqs. (1) and (2), the magmatic overpressure P_o_ in a dike with length L and width w is written as follows:

3 $${{\boldsymbol{P}}}_{{\boldsymbol{o}}}=\frac{{\boldsymbol{w}}{{\boldsymbol{\rho }}}_{{\boldsymbol{r}}}{{\boldsymbol{V}}}_{{\boldsymbol{s}}}^{2}}{{\boldsymbol{L}}(1-{\boldsymbol{\nu }})}$$

**Figure 4:**
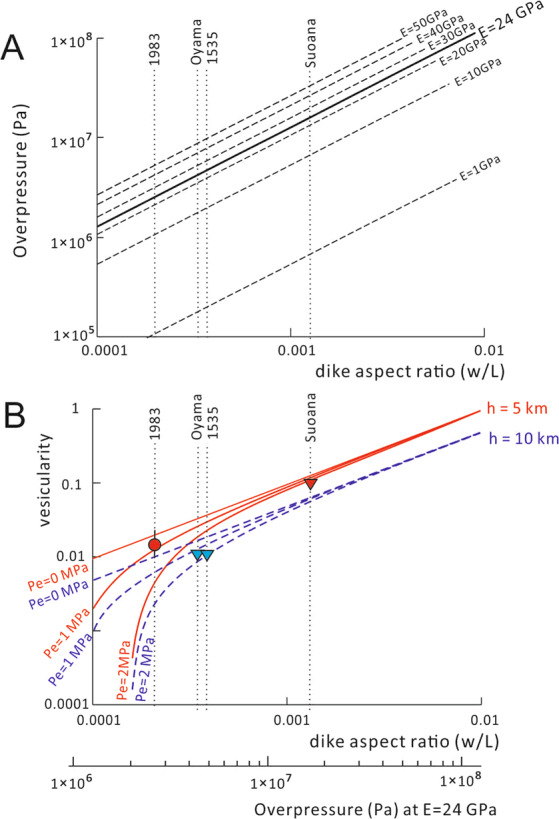
(**A**) Relationship between dike aspect ratio (w/L) and overpressure of the magma in the dike (P_o_) based on Eq. (3). Density of host rock (ρ_r_) is assumed as 2700 kg m^−3^. Variation of Young’s modulus of the host rock from 1 MPa to 50 MPa are shown. Broad solid line shows the relationship between w/L and Po at E = 24 GPa, suggested by the seismic wave Velocity of S-wave (V_s_) 1.9 km s^−1^ in the host rock. B: Relationship between dike aspect ratio (w/L) and vesicularity of magma (p) based on Eq. (6). The calculations are based on two different magma chamber depths (h = 5 km (solid red lines) and 10 km (broken blue lines)). The cases of P_e_ = 0, 1 and 2 MPa are shown for each case. Filled circles indicate the vesicularity in the chilled margin of the feeder dike (andesite) of the 1983 AD eruption. Filled triangles indicate the vesicularity of the least-vesiculated bombs of the Suoana (andesite: red), Oyama (basalt: blue) and 1535 (basalt: blue) eruptions, respectively. Scale at the bottom of the figure indicates the overpressure corresponding to the dike aspect ratio (w/L) shown in the horizontal axis of the figure based on Eq. (3) at E = 24 GPa.

Equation (3) shows that the magmatic overpressure in a dike can be evaluated based on aspect ratio of the dike w/L and the physical properties of the host rock (density, S-wave velocity, and Poisson’s ratio). The dike aspect ratios (w/L) of these feeder dikes are 12.5 × 10^−4^ for the Suoana eruption, 3.3 × 10^−4^ for the Oyama eruption, 3.7 × 10^−4^ for the 1535 AD eruption, and 2.0 × 10^−4^ for the 1983 AD eruption (Table 1, Fig. 4A). The estimated density of the magmas (ρ_0_) of these eruptions based on the whole-rock composition are 2650 kg m^−3^ for the andesite of the Suoana and the 1983 AD eruptions, and 2700 kg m^−3^ for the basalts of the Oyama and the 1535 eruptions (at a pressure of 0.1 MPa and assuming the absence of bubbles). Assuming that ρ_r_ = ρ_0_, then the density of the host rock (ρ_r_) can range between 2650–2700 kg m^−3^. We used a typical Poisson’s ratio (ν) of 0.25 in our model. As we could not measure the *in-situ* Young’s modulus of the host rock, we replaced Young’s modulus E with shear modulus G, which is the function of the S-wave velocity (V_s_) and density. The V_s_ in the host rock is assumed as 1.9 km s^−1^ based on the velocity model with a P-wave velocity of 3.3 km s^−1^ and a V_s_/V_p_ ratio of 0.57 in the shallow part of the volcanic edifice of Miyakejima, as proposed by^18^. We substituted these physical properties of the rocks into Eq. (2) and derived a value of Young’s modulus of the host rock of 24 ± 5 GPa. A wave velocity uncertainty of 10% was assumed.

After substituting these parameters into Eq. (3), the calculated overpressures for the Oyama, the 1535 and the 1983 AD eruptions are 4.2 ± 0.8, 4.7 ± 0.9 and 2.5 ± 0.9 MPa, respectively. A substantially higher overpressure of 16 ± 3 MPa was obtained from the feeder dike of the Suoana eruption (Fig. 4A).

## Vesicularity

Vesicularity of the magmas in the feeder dikes were estimated from the overpressure in the feeder dikes using Eq. (3). Magmatic overpressure P_o_ in a conduit is a sum of excess magmatic pressure P_e_ in the magma chamber, tectonic differential stress σ_d_, and a buoyancy force produced by the density contrast between the magma and the surrounding host rock.

4 $${{\boldsymbol{P}}}_{{\boldsymbol{o}}}={{\boldsymbol{P}}}_{{\boldsymbol{e}}}+({{\boldsymbol{\rho }}}_{{\boldsymbol{r}}}-{{\boldsymbol{\rho }}}_{{\boldsymbol{m}}}){\boldsymbol{gh}}+{{\boldsymbol{\sigma }}}_{{\boldsymbol{d}}}$$

where ρ_r_ and ρ_m_ are the density of the host rock and magma, respectively; g is the gravitational acceleration; h is the height of the conduit from the magma chamber; and σ_d_ is the difference between the maximum and minimum principal compressive stresses^1^. To simplify the model, we assume a wall rock with homogeneous density from the magma chamber to the edifice. As the volcanic edifice of Miyakejima is mainly composed of lava flows with mafic-intermediate composition, and the basement of the edifice also consists of an oceanic island arc, with volcanic and intrusive rocks of mafic-intermediate composition, the density variation between the edifice and the basement is negligible.

As magma decompresses during ascent, its vesiculation decreases its density. Assuming that the density of the vapor in bubbles is negligible, then the bulk density ρ_m_ of a vesicular magma is given as follows:

5 $${{\boldsymbol{\rho }}}_{{\boldsymbol{m}}}=(1-{\bf{p}}){{\boldsymbol{\rho }}}_{0}$$

where p is the vesicularity of magma. A magma chamber in equilibrium with its surrounding host rock at least partly denotes that the magma chamber resides at a depth of neutral buoyancy where the density of the magma ρ_m_ is equal to that of the surrounding host rock ρ_r_. The vesicularity of magma in a stable magma chamber is then essentially negligible, i.e., any exsolved volatiles contribute only to maintaining the equilibrium pressure. Otherwise, any additional gas exsolution in the chamber would promote pressure instabilities, potentially leading to unrest or chamber failure. Thus, the bulk density of the magma ρ_m_ in a stable chamber is equal to the density of non-vesiculated magma ρ_0_. Assuming ρ_r_ = ρ_0_, then the vesicularity of magma in a dike (p) can be expressed in Eq. (4) as follows:

6 $${\boldsymbol{p}}=\frac{{{\boldsymbol{P}}}_{{\boldsymbol{o}}}-({{\boldsymbol{P}}}_{{\boldsymbol{e}}}+{{\boldsymbol{\sigma }}}_{{\boldsymbol{d}}})}{{{\boldsymbol{\rho }}}_{0}{\boldsymbol{gh}}}$$

Depth to the magma chamber (h) was estimated from the petrological evidence in the samples deriving from each eruption. Based on the volatile concentrations in glass inclusions trapped in the phenocrysts of the products from the four studied eruption fissures, the basaltic magmas of Oyama and the 1535 AD eruptions were assumed to have been sourced from the chamber at a 10 km depth, and the andesitic magma of the Suoana and the 1983 AD eruptions were assumed to have been sourced from the chamber at a 5 km depth^10^.

The level of differential stress σ_d_ in the host rock controls, in part, the pattern of dike emplacement. The radial distribution of past eruption fissures in Miyakejima suggests a relatively small differential stress within the volcano. Considering this information, and with the knowledge that the investigated dikes were emplaced very near to the depth where the level of differential stress is often small, we neglected the effect of the differential stress in our model.

Excess magma pressure P_e_ is controlled by the tensile strength of the wall rock T_0,_ because the wall rock of a magma chamber or dike tip will rupture only when P_e_ reaches the tensile strength of T_0_^19^. Therefore, T_0_ gives the upper limit of P_e_. Experimental analyses of the rock samples and *in-situ* borehole tests have both indicated that the upper limit of representative tensile strengths of plutonic rocks can be as high as 10 MPa^20^. However, rupturing of the wall rock will occur with much lower excess pressures than laboratory-measured tensile strengths, owing to stress concentrations at the edge of the wall rock and/or local failures inside the wall rock^1^. When the magma in the dike has no buoyancy, overpressure P_o_ in the dike is equal to the excess pressure P_e_ in the magma chamber (Eq. (4)). As the estimated overpressures of the feeder dikes of Miyakejima range from 2.5 ± 0.5 to 16 ± 3 MPa, we assumed the upper limit of tensile strength for the crustal segment hosting Miyakejima to be 2 MPa (Table 1).

Substituting these parameters in Eq. (6), the mean vesicularities of the magmas in the feeder dikes are estimated as less than 2% for the Oyama, 1535 AD, and 1983 AD eruptions. The vesicularity of the feeder dike of the Suoana eruption was estimated as 10.5 ± 2.4%. If the tensile strength of the wall rock of the magma chamber is neglected and only the magma overpressure is supported by the buoyancy of the magma, then the vesicularities of the magmas in the feeder dikes are estimated as 1–2% for the Oyama eruption, the 1535 AD eruption and the 1983 AD eruption, and 10–15% for the Suoana eruption (Fig. 4B). These modeled value of vesicularities in these feeder dikes are consistent with the observed vesicularities of the chilled materials of these eruptions (Fig. 4B).

## Discussions

The aspect ratio of the feeder dikes of Miyakejima indicated a positive correlation between the magmatic overpressure within a feeder dike and eruption explosivity. As rupturing of pressurized bubbles in the magma generally controls the explosivity of an eruption, eruption of vesicular magmas with a high flux may result in an intensely explosive eruption. The presence of pressurized bubbles in magma also denotes magmatic overpressure in a feeder dike. Particularly, larger magmatic overpressure can be formed at the upper tip of dike in which bubbles concentrate. As magmatic overpressure within a feeder dike is a fundamental driving force of the magma flow toward the vent, a feeder dike with a higher overpressure can erupt magmas with an increased discharge rate. Magmatic overpressure also widens the feeder dike as shown by Eq. (1). As the magma flux through a tabular dike correlates with the length and square of the width of the dike, a larger magmatic overpressure results in an increased magma flux within the dike; this increase occurs through the widening of the feeder dike and increasing the flow speed.

Field observations in Miyakejima (Table 1) support this investigation; the feeder dike which formed explosive fire fountaining activity (Suoana eruption) has a larger dike aspect ratio, whereas the feeder dikes that formed effusive (Oyama eruption) and mild Strombolian (1535 and 1983 AD eruptions) eruptions have low dike aspect ratios. The vesicularities required to form the estimated overpressure in these feeder dikes were less than 2 vol.% for the Oyama, 1535 and 1983 eruptions, and ~10 vol.% for the Suoana eruption (Fig. 4B). The vesicularities of these feeder dikes are higher for the explosive eruption of Suoana than those of the less-explosive eruptions (of Oyama, 1535 and 1983). Considering their similar chemical compositions it is the difference in vesicularities which varies the amounts of overpressure in the feeder dikes and the explosivity of magmas. Heterogeneity of vesicularity within a feeder dike may also vary the explosivity of eruption. The concentration of bubbles at the tip of feeder dike can form higher overpressure locally at the dike tip which promotes intensive lava fountaining and high eruption rate at the opening of the eruption fissure, as observed during the 1983 eruption^15^.

A relatively low vesicularity in the feeder dikes obtained by Eq. (6), even for the dike of the explosive eruption, indicates effective outgassing from the conduit during the magma’s ascent. The initial water concentration in the magma, decompression, and the degree of outgassing from the magma into the surrounding host rock controls the vesicularity within the magma. Based on the water concentrations in glass inclusions trapped in phenocrysts, the initial water concentration in magmas within the magma chambers are estimated as >0.7 wt. % for andesite at 60–130 MPa and 1.9–3.5 wt% for primitive basalt at 250 MPa^10^. This initial water content clearly surpasses the amount required to achieve the estimated vesicularities for each feeder dike. This means that outgassing through the host rock controls both the vesicularity and the overpressure of magma in the feeder dike and, as result, suppresses the potential explosivity of fissure eruptions. As magma overpressure is controlled by outgassing from a feeder dike during ascent and prior to the onset of an eruption, monitoring of outgassing related to an ascending dike can provide clues as to the explosivity of an impending fission eruption.

**Figure 5:**
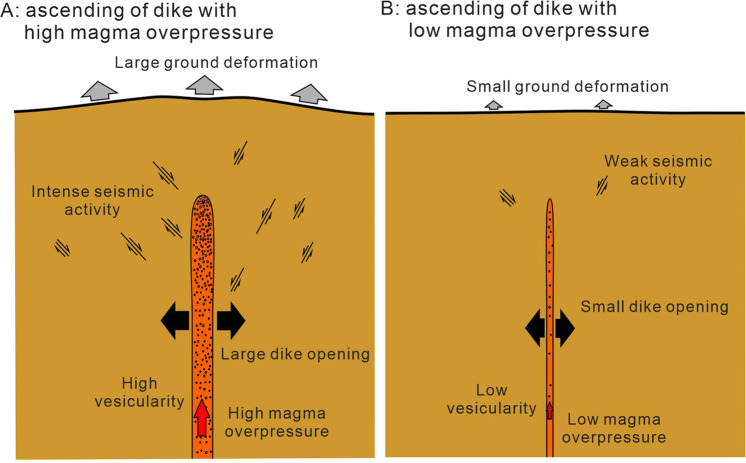
Illustration of the intrusion of dikes with high overpressure (**A**) and low overpressure (**B**). The emplacement of a dike with high magma overpressure due to high vesicularity causes intense seismic activity and deformation around the dike by the larger opening of dike (**A**), whereas a dike with lower magma overpressure causes weak seismic activity and deformation due to smaller dike opening. Explosive eruption is expected when the dike with high magma overpressure ruptures to the ground surface. Effusive or low explosive eruption is expected from the dike with low magma overpressure.

Our observations of dike geometry and the eruption styles in Miyakejima volcano indicate the impact of magmatic overpressure in the conduit on the explosivity of an eruption. Magmatic overpressure can be evaluated from the aspect ratio w/L of dikes. Using this model, we can forecast the potential explosivity of an impending fissure eruption based on the dike aspect ratio of an ascending feeder dike (Fig. 5). Suitably placed seismometers, GPS and tilt monitoring networks, as well as INSAR, can be used detect the length and opening of an ascending dike. Hence such techniques are crucial for the near-real time detection of magmatic overpressure within a dike and the potential explosivity forecasting of an impending eruption.

## Conclusions

Geological investigations of the conduit structures and erupted products of four historical fissure eruptions of Miyakejima volcano indicated a positive relationship between the estimated overpressure in the conduit and the relative explosivity of each eruption. This relationship is interpreted such that higher magmatic overpressures, caused by the buoyancy of vesicular magmas as a result of gas exsolution, widened the feeder dike and resulted in a higher discharge rate of bubble-rich magma. The estimated low vesicularity of magmas during the rising of feeder dikes indicate effective outgassing through the magma, or into the host rock, even for explosive eruptions. The model presented in this study provides a tool for detecting the development of both overpressure and gas exsolution in propagating dikes. Moreover, the model suggests the possibility of forecasting the explosivity of a fissure eruption by the detection of the aspect ratio of an ascending feeder dike. Real-time monitoring of the geometry of ascending dikes by suitably placed seismometers, GPS and tilt monitoring networks may realize the ability to forecast the explosivity of impending fissure eruptions.

## Methods

### Measurement of dike width

Overpressures within a feeder dike were estimated based on the dike aspect ratio (Kusumoto *et al*. 2013). We assumed that the horizontal length of the eruption fissure is the length of the feeder dike (L). The length of the eruption fissures (L) can be measured from the topographic and geological maps before the formation of the 2000 AD collapse caldera. The average width of the feeder dike in the outcrop represents the opening width of a dike (w). The width of each feeder dike (w) was measured either directly on the outcrop or derived from photographs where the dikes were not accessible. The feeder dikes of the Suoana, Oyama and the 1535 AD eruptions are exposed in the wall of the collapse caldera formed during the 2000 AD eruption^14^.

We used the photogrammetric method for determining dike widths on the caldera wall. The method used to determine the widths of the dikes is the same as described by^9^. Outcrop roughness and local irregularities in dike attitudes (possibly within 30° for each) are the most likely sources for measurement error, which we estimated at less than 20%.

The feeder dike of the 1983 eruption is instead exposed in the wall of a maar that was formed by phreatomagmatic explosions in the later stage of the eruption^16^. The width and thickness of the feeder dike were measured directly on the outcrop.

### Estimation of magma density

The magma density was estimated by the whole-rock chemical composition and magmatic temperature. The whole-rock chemical compositions of the erupted magmas were determined with an X-ray fluorescence (XRF) spectrometer at the Geological Survey of Japan, using a glass-bead method with a 1:10 ratio of dilution^21^. We measured eight samples from the ejecta of the initial phase of the Suoana eruption, six samples from the Oyama eruption, four samples from the 1535 AD eruption, and four from the 1983 AD eruption (Table S1 in the Supplementary material). The averaged whole-rock chemical compositions of each eruption were used for calculating the magma density. The densities of the magmas of the eruptions were calculated from the whole-rock chemical compositions using the method of^22^. The pressure and temperature conditions for the density estimations were assumed at 0.1 MPa and 1100 °C, respectively.

## Supplementary information

Supplementary information.

Supplementary Figure S1.

Supplementary Table S1.
