# Supplementary material for: Magmatic overpressures, volatile exsolution and potential explosivity of fissure eruptions inferred via dike aspect ratios
Source: Sci Rep. 2020 Jun 10;10:9406. doi: 10.1038/s41598-020-66226-z (PMC7287056; doi:10.1038/s41598-020-66226-z)
Supplement: Supplementary file 1 — Supplementary information. [file 41598_2020_66226_MOESM1_ESM.docx]

**Supplementary Information**

**Magmatic overpressures, volatile exsolution and potential explosivity of fissure eruptions inferred via dike aspect ratios**

Nobuo Geshi, John Browning, Shigekazu Kusumoto

Figure S1

Feeder dikes and their eruptive products outcropping in the wall of the 2000 AD caldera. A: Vent system of the Suoana eruption. Broken white lines indicate the deposit of agglutinate from the Suoana crater in the early stage of the eruption. B: Vent system of the Oyama eruption. Broken white lines indicate the pile of lavas erupted from the feeder dike. C: Vent system of the 1535 AD eruption. Scoria cone fed by the feeder dike is highlighted by the broken white lines. Feeder dike of each eruption fissure is indicated by white arrow at the base of the outcrop.

Table S1

Whole-rock compositions of the magmas of the fissure eruptions.
