# Supplementary figures and images for: Magmatic overpressures, volatile exsolution and potential explosivity of fissure eruptions inferred via dike aspect ratios
Source: Sci Rep. 2020 Jun 10;10:9406. doi: 10.1038/s41598-020-66226-z (PMC7287056; doi:10.1038/s41598-020-66226-z)

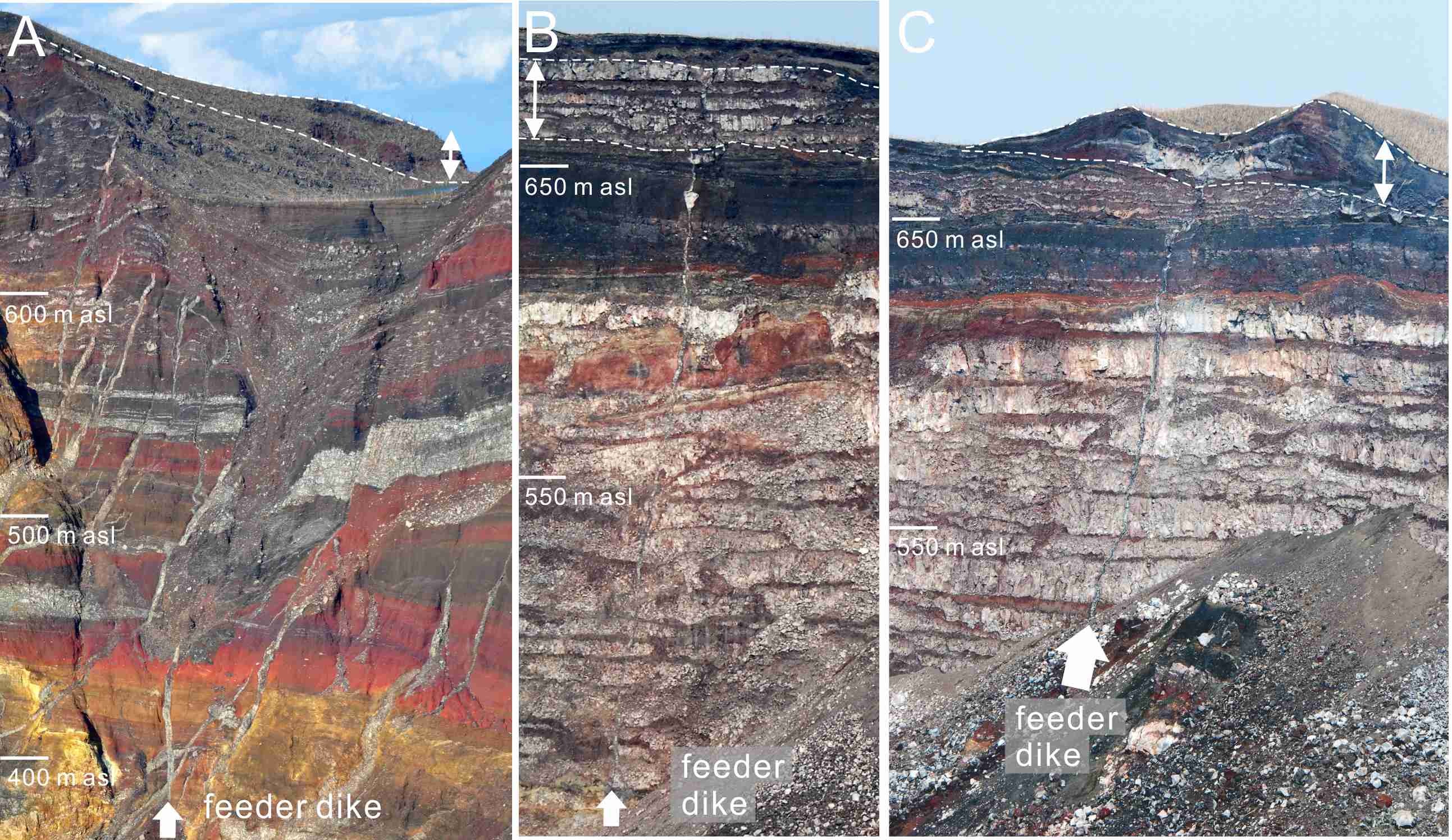

Supplement: Supplementary file 2 — Supplementary Figure S1. [file 41598_2020_66226_MOESM2_ESM.jpg]

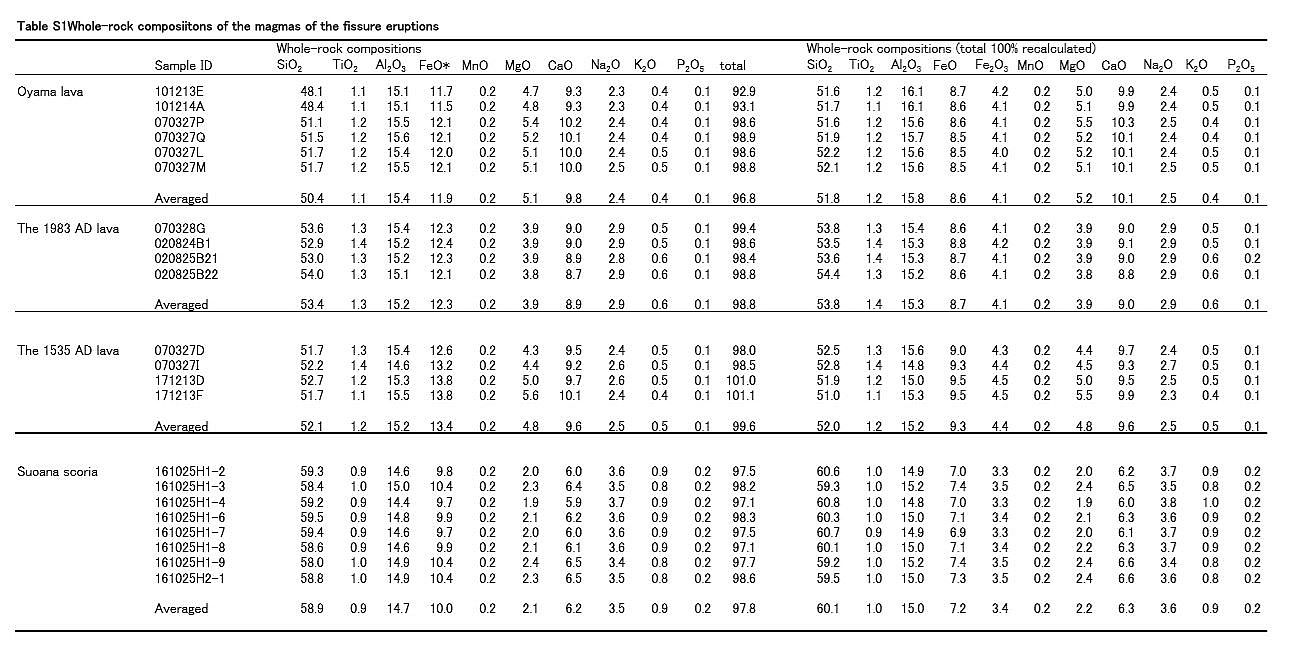

Supplement: Supplementary file 3 — Supplementary Table S1. [file 41598_2020_66226_MOESM3_ESM.jpg]
